# Phosphorylation-dependent stabilization of MZF1 upregulates N-cadherin expression during protein kinase CK2-mediated epithelial-mesenchymal transition

**DOI:** 10.1038/s41389-018-0035-9

**Published:** 2018-03-13

**Authors:** Hyeonseok Ko, Seongrak Kim, Kyungmi Yang, Kunhong Kim

**Affiliations:** 1 Laboratory of Molecular Oncology, Cheil General Hospital & Women’s Healthcare Center, Dankook University College of Medicine, Jung-gu, Seoul 04619 Korea; 2 Department of Biochemistry and Molecular Biology, Yonsei University College of Medicine, 50-1 Yonsei-ro, Seodaemun-gu, Seoul 03722 Korea; 3 Integrated Genomic Research Center for Metabolic Regulation, 50-1 Yonsei-ro, Seodaemun-gu, Seoul 03722 Korea

## Abstract

Epithelial-mesenchymal transition (EMT) is a critical process in invasion and metastasis of cancer cells. E-cadherin to N-cadherin switching is considered a molecular hallmark of EMT. Recently, we reported that increased CK2 activity fully induces E-cadherin to N-cadherin switching, but the molecular mechanisms of N-cadherin upregulation are unknown. In this study, we examined how N-cadherin is upregulated by CK2. *N-cadherin* promoter analysis and ChIP analysis identified and confirmed myeloid zinc finger 1 (MZF1) as an *N-cadherin* transcription factor. Molecular analysis showed that MZF1 directly interacts with CK2 and is phosphorylated at serine 27. Phosphorylation stabilizes MZF1 and induces transcription of *N-cadherin*. *MZF1* knockdown (MKD) in N-cadherin-expressing cancer cells downregulates *N-cadherin* expression and reverts the morphology from spindle and fibroblast-like to a rounded, epithelial shape. In addition, we showed that that MKD reduced the motility and invasiveness of N-cadherin-expressing cancer cells. Collectively, these data indicate that N-cadherin upregulation in CK2-mediated E-cadherin to N-cadherin switching is dependent on phosphorylation-mediated MZF1 stabilization. CK2 could be a good therapeutic target for the prevention of metastasis.

## Introduction

The term epithelial-mesenchymal transition (EMT) describes a biological process during which cells lose their epithelial characteristics and acquire properties of mesenchymal cells through multiple biochemical changes^1^. The transitioned cells are characterized by a loss of epithelial cell polarity, disassembly of cell-cell junctions, and increased cell motility^2,3^. EMT occurs during many biological processes of normal and diseased states, such as implantation, embryogenesis, organ development, wound healing, tissue regeneration, organ fibrosis, and tumor progression^4^.

E-cadherin to N-cadherin switching, which often occurs during EMT, refers to the replacement of E-cadherin expression with N-cadherin^5–10^ and is a molecular hallmark of EMT^4^. The cadherin family mediates calcium-dependent, homotypic cell-cell adhesion and plays important roles in the maintenance of normal tissue architecture^11,12^. The classical cadherins, E-cadherin, N-cadherin, and P-cadherin, are expressed in specific cells and tissues, and during specific developmental stages. E-cadherin is the major cadherin in polarized epithelial cells, whereas N-cadherin is expressed mainly in mesenchymal cells^13^. Transcriptional repression of *E-cadherin* is a major molecular mechanism for the loss of E-cadherin expression during cadherin switching^14^. *E-cadherin* transcriptional repressors have been extensively studied in the last decade, and they include the Snail superfamily of zinc-finger transcriptional repressors, Snail1^15,16^ and Snail2 (also known as Slug)^17,18^, the ZEB family of transcription factors, ZEB1 (also known as TCF8 and δEF1) and ZEB2 (also known as ZFXH1B and SMAD interacting protein 1; SIP1)^19,20^, bHLH factors, Twist1, E47 (also known as TCF3), and TCF4 (also known as E2-2)^21,22^. In contrast to E-cadherin, little is known about the regulatory mechanisms pertaining to the upregulation of N-cadherin^23^.

Protein kinase CK2 is a constitutively active, growth factor-independent serine/threonine protein kinase with key roles in cell cycle control, cellular differentiation, proliferation, and regulation of apoptosis^24–26^. Elevated levels of CK2 expression or activity have been reported in many cancer types^25,27–29^. In addition, overexpression of the CK2α catalytic subunit is known to induce tumor formation^28^, indicating a protumorigenic role for CK2. Recently, we reported that increased CK2 activity fully induces E-cadherin to N-cadherin switching and CK2 activity is required for the maintenance of N-cadherin expression^30^, confirming the role of CK2 in EMT.

While CK2 fully induces E-cadherin to N-cadherin switching, the molecular mechanisms for N-cadherin upregulation during switching remain largely unknown. Therefore, in this study, we examined how N-cadherin is upregulated during CK2-mediated E-cadherin to N-cadherin switching.

## Results

### Identification of MZF1 as an *N-cadherin* transcription factor

**Fig. 1:**
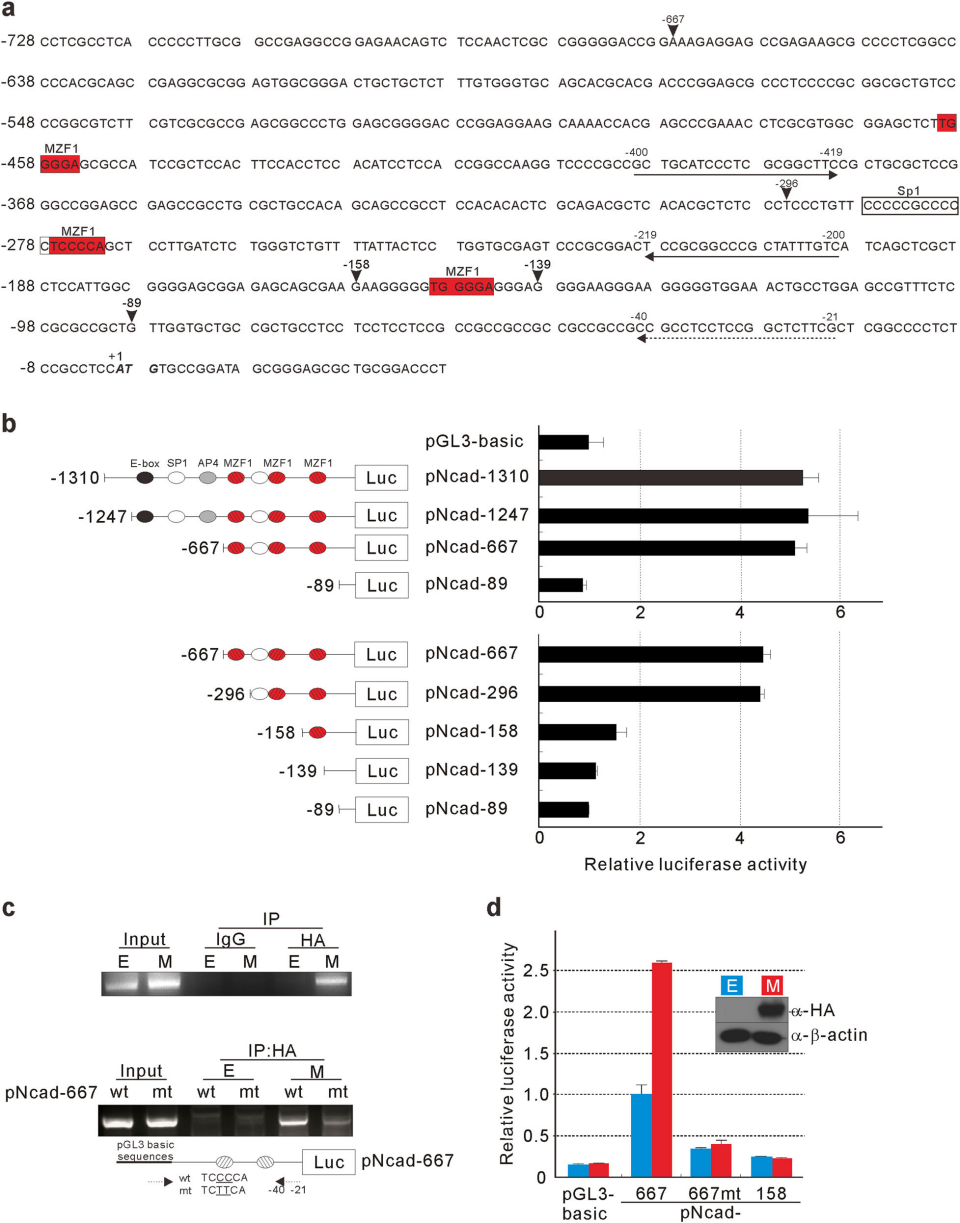
Identification of MZF1 as an *N-cadherin* transcription factor. **a** Nucleotide sequences of the 5′-flanking region and exon 1 of the human *N-cadherin* gene^31^. The ATG start codon is shown in bold, italicized font and is designated as +1. The arrowheads indicate the nucleotides at which various deletion constructs of the *N-cadherin* promoter were created. Putative binding sites for MZF1 are shown in red boxes. **b** *N-cadherin* promoter activity analysis. HCE4 cells were transiently transfected with pNcad-1310 or its 5′-serial deletion constructs. Normalized luciferase activities are shown as mean ± SD of triplicate samples and are expressed as fold-increase relative to the luciferase activity from pGL3-basic. E-box, SP1, AP4, and MZF1-binding sites are shown. **c** Chromatin immunoprecipitation (ChIP) analysis of MZF1 binding to the *N-cadherin* promoter. HCE4 cells were transfected with pSG5-HA (E) or pSG5-HA-MZF1 (M); chromatin was immunoprecipitated with the indicated antibodies (top). The sequences of the forward (−400 to −419) and reverse (−219 to −200) primers for ChIP analysis are underlined and indicated with an arrow in (**a**). Sequences in the distal MZF1-binding site, TCCCCA (wt), were mutated into TCTTCA (mt) by site-directed mutagenesis of pNcad-667. Cells were cotransfected with either pSG5-HA-MZF1 and pNcad-667wt or pSG5-HA-MZF1 and pNcad-667mt; chromatin was immunoprecipitated with the indicated antibodies (bottom). The sequences of the reverse (−21 to −40) primer for ChIP analysis are underlined and indicated with a dashed arrow in (**a**). The sequences of the forward primer are present in pGL3 basic. **d** Effect of exogenous MZF1 on *N-cadherin* promoter activity. HCE4 cells were cotransfected with pGL3-basic, pNcad-667, pNcad-667mt, or pNcad-158 plus either pSG5-HA (E) or pSG5-HA-MZF1 (M). Western blot analysis of exogenous HA-tagged MZF1 expression is shown in the inset. Normalized luciferase activities are shown as mean ± SD of triplicate samples and are shown as fold-increase or fold-decrease relative to the activity measured in cells cotransfected with pNcad-667 and pSG5-HA

Previously, it was reported that MZF1 regulates *N-cadherin* promoter in osteoblasts^31^. To identify a transcription factor for regulating *N-cadherin* expression in cancer cells, the human *N-cadherin* promoter region (−1310 to +190) was cloned into the pGL3 basic plasmid (pNcad-1310), and serial deletion constructs were generated (Fig. 1b). The *N-cadherin* promoter reporter assay was performed in mesenchymally transitioned HCE4 cells^30^. *N-cadherin* promoter activity was reduced after deletion of the 5′ end of the promoter to position −158 (Fig. 1b). The promoter sequences of the human *N-cadherin* gene were analyzed and two MZF1-binding sites were found in pNcad-296 (Fig. 1a, b). One of the sites was a proximal MZF1 binding site located between the −150 and −145 positions (TGGGGA, ); this site was previously reported as an important site for *N-cadherin* expression in osteoblasts^31^, and the other site was a distal MZF1 binding site located between positions −277 to −272 (TCCCCA, ) in an inverted orientation. The distal site could be the binding site for MZF1 because it was located between positions −296 and −158 (Fig. 1a, b). ChIP assays confirmed the results from the reporter assay showing that MZF1 specifically binds to the distal MZF1 binding site (Fig. 1c). Consistent with this result, MZF1 overexpression significantly increased the reporter activity of pNcad-667 but not that of pNcad-667mt or pNcad-158 (Fig. 1d).

### Confirmation of MZF1 as an N-cadherin transcription factor

**Fig. 2:**
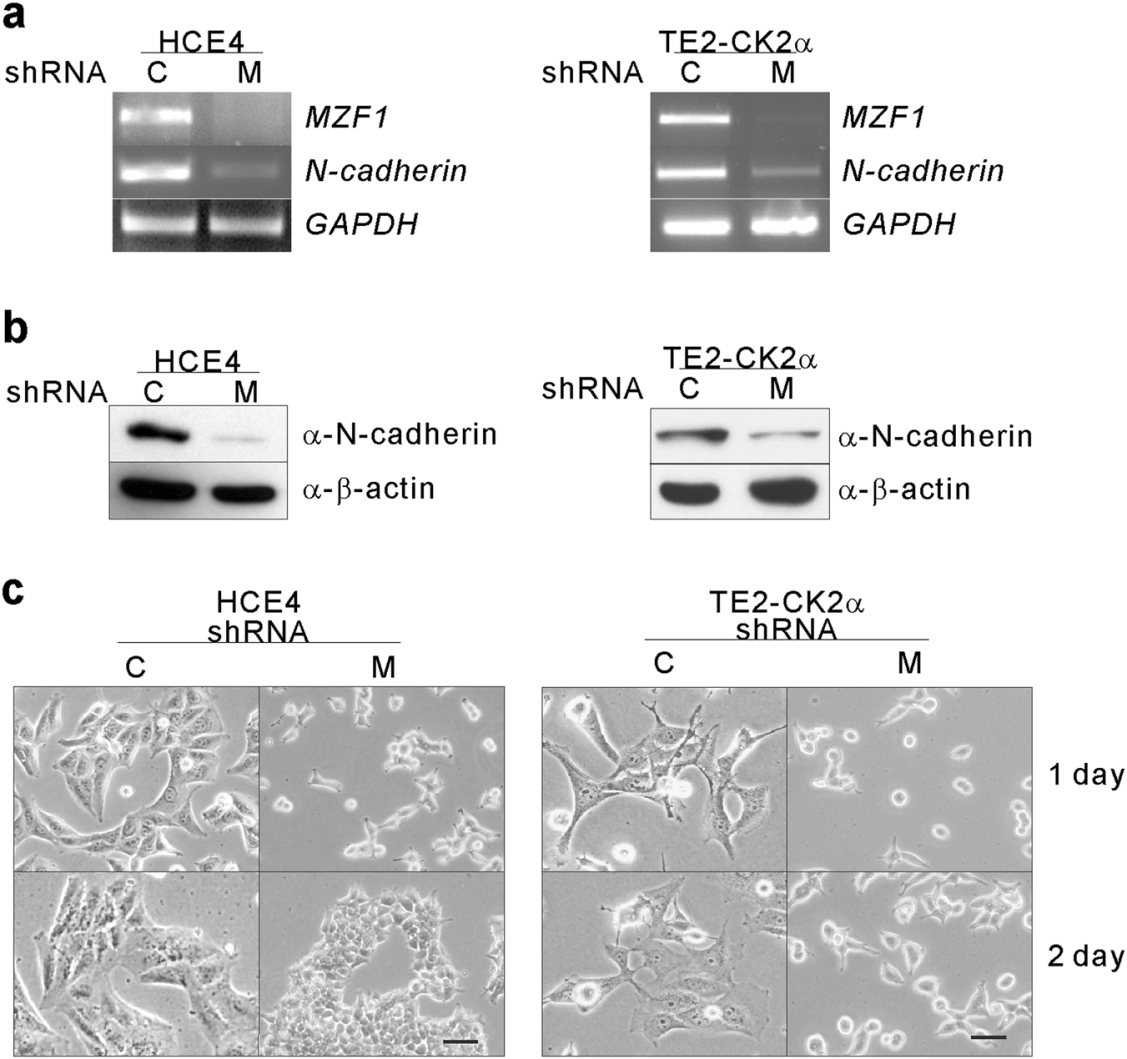
MZF1 is an *N-cadherin* transcription factor. **a** Effect of MKD on gene expression using semi-quantitative RT-PCR for indicated genes; *GAPDH* was used as an internal control. HCE4 (left) and TE2-CK2α (right) scrambled shRNA: C and shRNA against MZF1: M. **b** Western blot analyses were performed with the indicated antibodies. β-actin was used as a loading control for total cell lysates. **c** Effect of shRNA-mediated MKD on morphology. Scale bars, 50 μm

To confirm whether MZF1 is a transcription factor for *N-cadherin* expression in mesenchymally transitioned cancer cells, we generated stable *MZF1* knockdown (MKD) HCE4 cells and TE2-CK2α^30^ cells using short hairpin RNA (shRNA). Because no good MZF1 antibody (Ab) is commercially available, RT-PCR was performed to confirm MKD. We found that *MZF1* expression was knocked down by the shRNA, and MKD resulted in downregulation of *N-cadherin* expression (Fig. 2a). Consistent with the RNA results, N-cadherin expression was downregulated, as shown by western blot analysis (Fig. 2b). In addition, MKD caused morphologic changes to mesenchymally transitioned cancer cells from spindle and fibroblast-like to a rounded, epithelial shape (Fig. 2c).

### MZF1 is phosphorylated by CK2

**Fig. 3:**
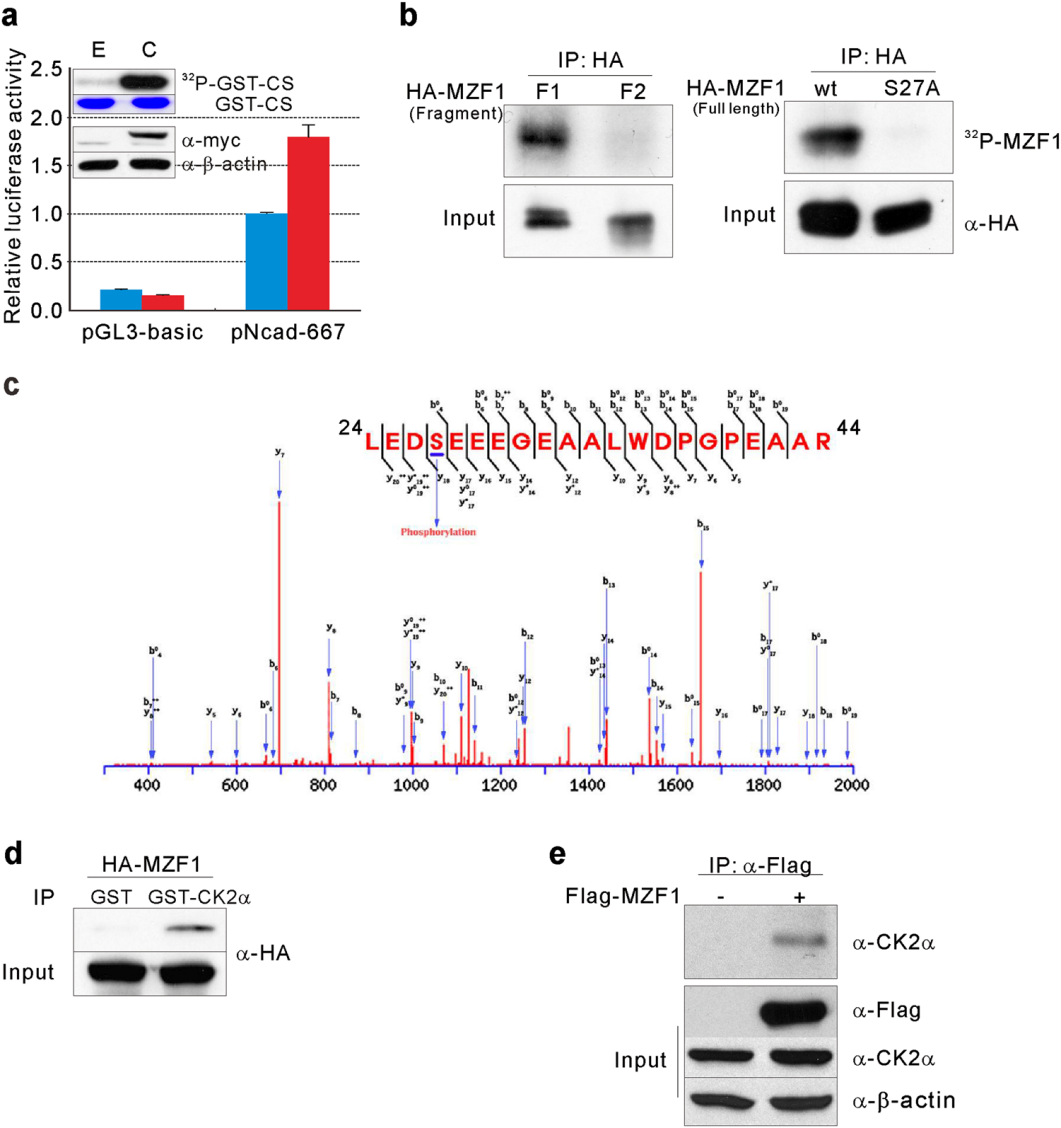
MZF1 is phosphorylated by protein kinase CK2. **a** Effects of increased CK2 activity on *N-cadherin* promoter activity. Promoter activities of pGL3-basic or pNcad-667 were measured in HEK293 cells expressing either pCMV-myc (E) or pCMV-myc-CK2α (C). Results of western blot analysis for the exogenous expression of myc-CK2α and in vitro kinase assays for intracellular CK2 activity are shown in the inset. GST-CS represents input GST-CS stained with Coomassie brilliant blue. ^32^P-GST-CS represents phosphorylated GST-CS. Normalized luciferase activities are shown as mean ± SD for triplicate samples and are shown as fold-increase or fold-decrease relative to the activity from cells cotransfected with pNcad-667 and pCMV-myc. **b** CK2 phosphorylates MZF1 at serine 27. HEK293 cells were transfected with HA-MZF1 F1 (amino acid residues 1 to 240 of MZF1) or F2 (amino acid residues 120–360 of MZF1) (left) or with full-length MZF1 wt or MZF1 S27A (right). Exogenously expressed MZF1 variants were immunoprecipitated and used as substrates for in vitro kinase assays. ^32^P-MZF1 represents HA-tagged MZF1 phosphorylated by CK2. **c** Identification of serine 27 as a CK2 phosphorylation site in MZF1 using mass spectrometry. Fragmentation spectrum (with *b* and *y* ions indicated) of a peptide spanning from amino acid 24 to 44 showing a phosphorylated serine at position 27 in MZF1. **d** Interaction between MZF1 and GST-tagged CK2α in vitro. Protein lysates isolated from HA-tagged MZF1-overexpressing HEK293 cells were mixed with human recombinant CK2 (GST-CK2α). Immunoprecipitation with a GST-specific Ab was followed by western blot analysis using anti-HA Ab. **e** Interaction between exogenous MZF1 and endogenous CK2α. HEK293 cells were transfected with Flag-MZF1, and lysates were immunoprecipitated with anti-Flag Ab (α-Flag) followed by western blot analysis using anti-CK2α Ab. Results of western blot analysis of total cell lysates are labeled as ‘Input’

We hypothesized that CK2 might upregulate *N-cadherin* expression through phosphorylation-dependent MZF1 modulation. First, we examined whether increased CK2 activity could affect *N-cadherin* promoter activity in cancer cells. Exogenous overexpression of the CK2α catalytic subunit increased CK2 activity and enhanced the promoter activity of pNcad-667 (Fig. 3a). Then, we examined whether CK2 could phosphorylate MZF1. Eleven potential CK2 phosphorylation sites^32^ in MZF1 were predicted by the KinasePhos2.0^33^ program. All potential phosphorylation sites were located within the first 360 amino acids of MZF1. Two HA-tagged MZF1 fragment expression constructs were generated and expressed. Fragment 1 (F1) contained amino acids 1–240, and F2 contained amino acids 120–360. Protein lysates were immunoprecipitated and processed for the in vitro kinase assay to detect CK2-dependent phosphorylation of these peptides. F1, but not F2, was phosphorylated (Fig. 3b, left), suggesting that at least one CK2 phosphorylation site is located within amino acids 1–120. As serine 27 and serine 111 were two predicted phosphorylation sites within amino acids 1–120, these serines were replaced with alanine by site-directed mutagenesis. Immunoprecipitation followed by in vitro kinase assay showed that full-length MZF1 27A showed no phosphorylation, indicating that serine 27 of MZF1 is the phosphorylation site (Fig. 3b, right). Mass spectrometric analysis also showed serine 27-phosphorylation of MZF1 by CK2 (Fig. 3c). In addition, a GST pulldown assay showed that CK2α interacted directly with MZF1 (Fig. 3d); the interaction between exogenous MZF1 and endogenous CK2α was confirmed in 293 cells (Fig. 3e).

### Effect of CK2-mediated phosphorylation on MZF1

**Fig. 4:**
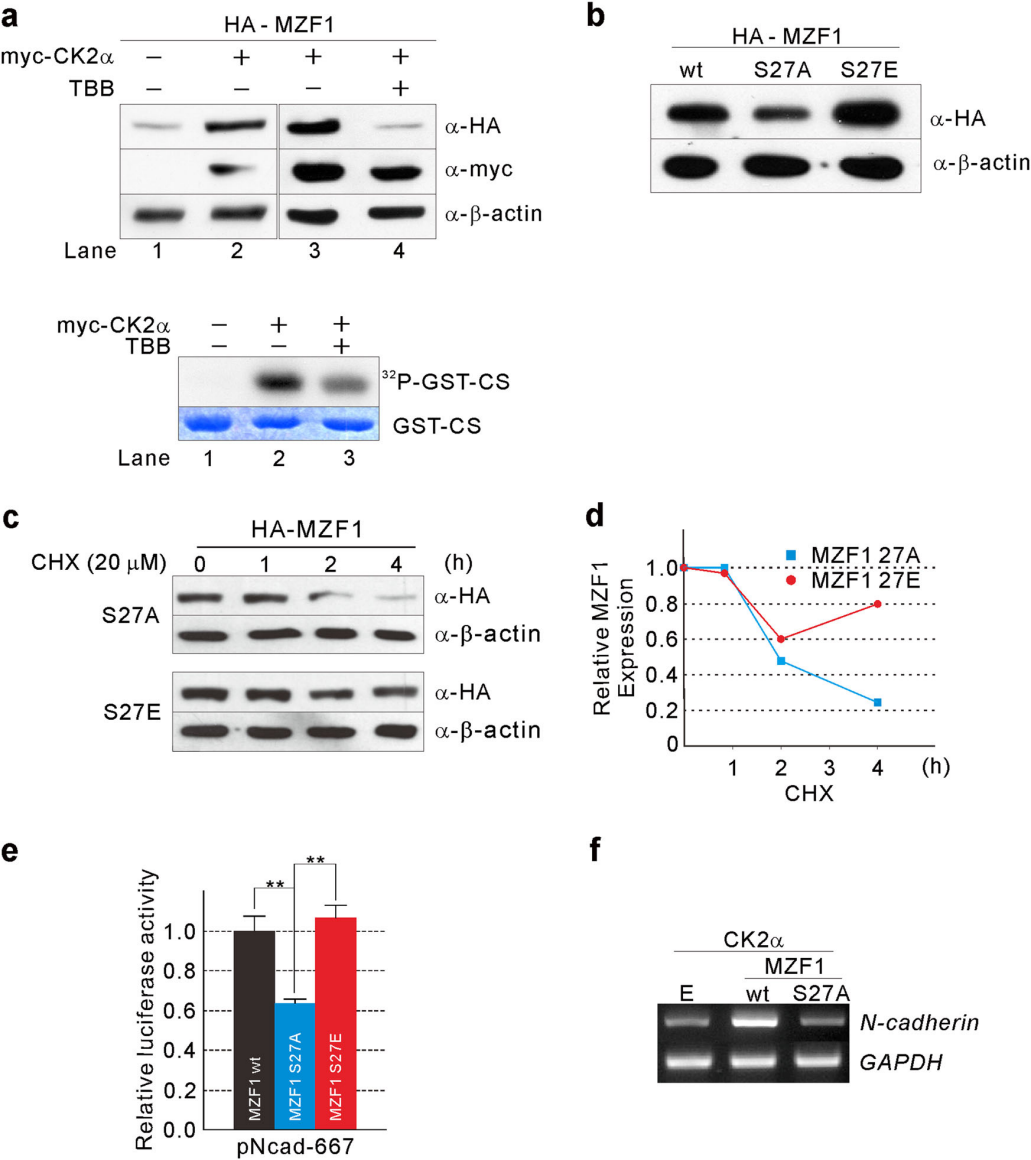
Effect of CK2-mediated phosphorylation on MZF1. **a** CK2 activity-dependent MZF1 expression. TE2 cells were cotransfected with HA-MZF1 and myc-CK2α expression vector. These cells were subsequently treated or not with a CK2 inhibitor, TBB (20 μM), for 24 h. Western blot analysis was performed with the indicated antibodies (top), and in vitro kinase assays were performed (bottom). **b** Expression of each MZF1 phospho-mutant. HCE4 cells were transfected with MZF1 wt, MZF1 S27A, or MZF1 S27E. Cell lysates were prepared from each transfectant followed by western blot analysis with the indicated antibodies. **c** MZF1 stabilization by phosphorylation. HEK293 cells transfected with S27A or S27E were treated with cycloheximide (CHX; 20 μM) and harvested after the indicated time intervals. Lysates were prepared, and western blot analysis was performed with anti-HA Ab. β-actin was used as a loading control. **d** The relative expression levels of MZF1 were plotted against treatment time with CHX. **e** Effect of MZF1 wt or each phospho-mutant on *N-cadherin* promoter activity. Normalized luciferase activities are shown as mean ± SD of triplicate samples and are expressed as fold-increase or fold-decrease relative to the activity from HCE4 cells cotransfected with pNcad-667 and MZF1 wt. **P < 0.01. **f** Functional effect of MZF1 expression on *N-cadherin* transcription. TE2 cells cotransfected with myc-CK2α and empty vector (E), MZF1 wt, or MZF1 S27A. Semi-quantitative RT-PCR was performed. *GAPDH* was used as an internal control

To examine the effect of CK2-mediated phosphorylation on MZF1, TE2 cells, which have low CK2 activity^25^, were cotransfected with HA-MZF1 and myc-CK2α expression vectors. The MZF1 level was elevated in the cells (Fig. 4a, top; lane 2 vs 1) with increased CK2 activity (Fig. 4a, bottom; lane 2 vs 1). When CK2 activity was inhibited by tetrabromo benzotriazole (TBB) (Fig. 4a, bottom; lane 2 vs 3), a pharmacologic CK2 inhibitor, expression of MZF1 returned to the level of the control (Fig. 4a, top; lane 3 vs 4). To confirm the correlation between the expression level of MZF1 and CK2 activity, we used the non-phosphorylatable MZF1 S27A mutant that could mimic MZF1 in cells with extremely low CK2 activity and the phosphomimetic MZF1 S27E that could mimic MZF1 in cells with extremely high CK2 activity. HCE4 cells, which have high CK2 activity^25^, were transfected with MZF1 wt, MZF1 S27A, or MZF1 S27E expression vectors. Western blot analysis showed that the expression level of MZF1 was correlated with its phosphorylation status (Fig. 4b). To examine whether CK2 activity-dependent increases in MZF1 expression are due to phosphorylation-dependent MZF1 stabilization, we transfected HEK293 cells with MZF1 S27A or S27E and treated the cells with cycloheximide. Western blot analysis revealed that CK2-mediated phosphorylation stabilizes MZF1 protein expression (Fig. 4c, d). Consistent with the results from Fig. 4b, luciferase reporter analysis showed that *N-cadherin* promoter activity was correlated with the MZF1 expression level (Fig. 4e). Finally, we examined the functional effect of MZF1 expression on *N-cadherin* transcription by semi-quantitative RT-PCR in TE2 cells. We found that CK2 could upregulate *N-cadherin* transcription in the presence of MZF1 wt, but not in the presence of MZF1 S27A (Fig. 4f).

### MZF1 effect on motility and invasiveness

**Fig. 5:**
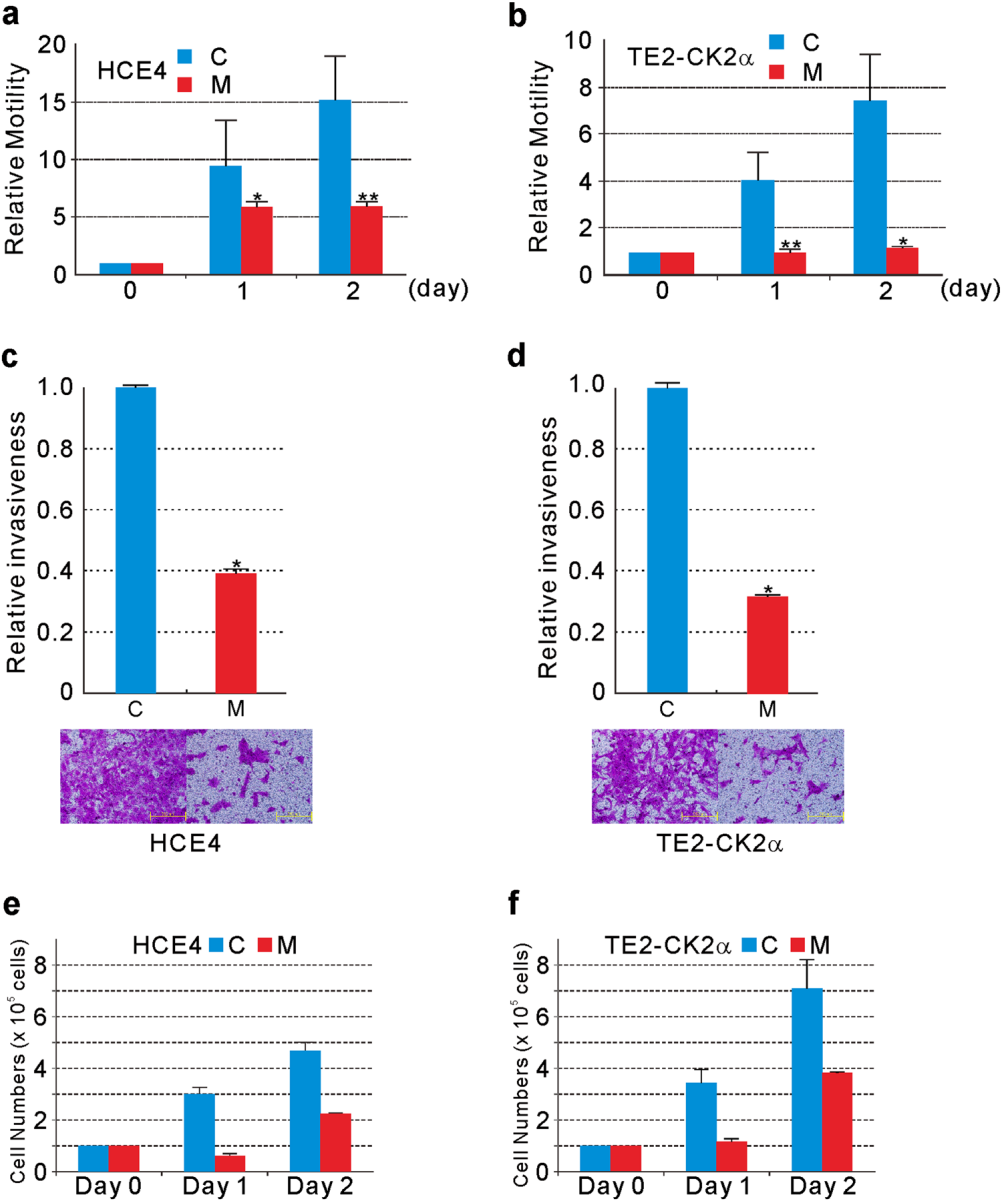
Effect of MKD on motility, invasiveness, and proliferation. Effect of MKD on motility of HCE4 cells (**a**) or TE2-CK2α cells (**b**). Motility was measured with the OrisTM Cell Migration Assay kit. *P < 0.05, **P < 0.01 compared to the C. Effect of MKD on invasion of HCE4 cells (**c**) or TE2-CK2α cells (**d**). The cells were incubated for 48 h in the upper chambers coated with Matrigel. Data represent the mean ± SD of one experiment performed in triplicate. Similar results were obtained from two independent experiments. *P < 0.05 compared to the C. Effect of MKD on proliferation of HCE4 cells (**e**) or TE2-CK2α cells (**f**). Cells were counted at 24 and 48 h after seeding. Data represent the mean ± SD of one experiment performed in triplicate. Similar results were obtained from two independent experiments. C represents scrambled shRNA and M represents *MZF1* shRNA, respectively

N-cadherin-expressing cancer cells are motile, invasive, and metastatic^34,35^. MKD in mesenchymally transitioned HCE4 or TE-CK2α cells resulted in decreased motility (Fig. 5a, b, and Supplementary Figure 2) and invasiveness (Fig. 5c, d).

## Discussion

EMT is a complex molecular event that regulates cell motility, invasiveness, and metastatic tumor spread. Expression of N-cadherin, with the concurrent loss of E-cadherin, is a representative molecular change in the EMT process. This study shows that CK2-mediated MZF1 stabilization could upregulate transcription of *N-cadherin*. We found that MZF1 was phosphorylated at serine 27 by CK2, and this modification increased the stability of MZF1.

Our recent report showed that E-cadherin to N-cadherin switching is achieved only by increasing CK2 activity using esophageal cancer cell lines^30^. We also could demonstrate CK2-mediated E-cadherin to N-cadherin switch in human lung cancer cells and human colon cancer cells (data not shown). To reprogram gene expression during the switch, CK2 may modulate transcription factors responsible for *E-cadherin* downregulation and *N-cadherin* upregulation. The notion that MZF1 may be involved in *N-cadherin* expression is hinted by a previous report in human osteoblasts^31^. However, this idea was not fully substantiated in cancer. To identify the transcription factor for CK2-mediated *N-cadherin* upregulation in cancer cells, *N-cadherin* promoter deletion analysis was performed. In osteoblasts, two regions (−335 to −214; −214 to −74) were reported to be essential for *N-cadherin* transcription, one for the putative Sp1 binding site (CCCCCGCCCCC −288 to −278) and the other for the MZF1 binding site (TGGGGA located between −150 and −145; proximal MZF1 binding site)^31^. In mesenchymally transitioned cancer cells, we found a region (−296 to −158) that showed the highest *N-cadherin* promoter activity, and it contained an additional MZF1 binding site (TCCCCA located between −277 and −272; distal MZF1 binding site) that was not reported previously^31^. Consistent with the results from our promoter reporter assay, ChIP analysis showed the strong binding of MZF1 to the distal MZF1 binding site (Fig. 1c). When the distal MZF1 binding site was mutated (TCCCCA to TCTTCA), the binding became reduced even in the presence of intact proximal MZF1 binding site, indicating that MZF1 binds mainly to the distal MZF1 binding site in cancer cells (Fig. 1c). Overexpression experiments showed MZF1 could increase reporter activity with wt *N-cadherin* promoter but no activity increase was observed with mutant *N-cadherin* promoter that has mutations in distal MZF1 binding site. It was concluded that, unlike osteoblast, MZF1 binds preferentially to the distal MZF1 binding site of *N-cadherin* promoter in cancer cells. Since *N-cadherin* transcription was suppressed by MKD in TE2-CK2α cells, we concluded that MZF1 is a transcription factor responsible for CK2-mediated *N-cadherin* upregulation in cancer cells (Fig. 2a, b).

How E-cadherin is downregulated during CK2-mediated cadherin switch? The transcription factor for *E-cadherin* downregulation is Snail. CK2 phosphorylates Snail at serine 92 for stabilization^36^. CK2 also phosphorylates β-catenin at threonine 393 for stabilization^37,38^. Stabilized β-catenin then upregulates Axin2 expression, increased Axin2 shuttles GSK3β out from the nucleus, and thus, nuclear Snail can be no longer degraded^39^. We had examined the expression of β-catenin, Snail, or *Axin2* according to the CK2 activity of cancer cells. The expressions of Snail, β-catenin, and *Axin2* were higher in cells with high CK2 activity (HCE4) than cells with low CK2 activity (TE2 or HCE4 cells treated with TBB) (Supplementary Figure 3).

N-cadherin-expressing cancer cells are motile, invasive, and metastatic^34,35^. In agreement with previous observations, we found that cancer cells were motile as long as N-cadherin was expressed^30^. Downregulation of N-cadherin in response to MKD resulted in decreased motility (Fig. 5a, b). In addition to motility, the invasiveness of cancer cells was dictated by the type of cadherin expressed (Fig. 5c, d)^30^. Our findings are consistent with reports that migration and invasion of cancer cells depend on the expression level of MZF1^40,41^.

In summary, our results show that CK2 is able to phosphorylate and stabilize MZF1, thereby upregulating *N-cadherin* transcription during E-cadherin to N-cadherin switch in cancer cells. Our results suggest that CK2 could be a good therapeutic target that could inhibit metastasis of cancer cells.

## Materials and methods

### Cell culture and reagents

The human esophageal cancer cell lines TE2, TE2-CK2α, and HCE4, and the human embryonic kidney cell line HEK293 were cultured in Dulbecco’s modified Eagle’s medium (DMEM) (Gibco Laboratories, Gaithersburg, MD, USA) supplemented with 10% fetal bovine serum (FBS) (Gibco). The CK2 inhibitor, TBB (Sigma-Aldrich, St. Louis, MO, USA), was prepared as a 20 mM stock in DMSO (Sigma-Aldrich).

### Western blot analysis

Western blot analysis was performed as previously described^30^. Blotted membranes were immunostained with antibodies specific for the following antigens: Myc tag (Cell Signaling Technology Inc., Beverly, MA), HA tag, β-actin (Sigma-Aldrich), E-cadherin, and N-cadherin (Invitrogen Co., Carlsbad, CA). Signals were developed using Lumi-Light Western Blotting Substrate (Roche, Indianapolis, IN, USA), according to the manufacturer’s protocol.

### Dual-luciferase reporter assay

The indicated plasmids were transfected into the indicated cell lines to examine *N-cadherin* or *Snail* promoter activity. The pRL-TK plasmid was cotransfected in all of the experiments to normalize luciferase activity. Forty eight hours after transfection, cell lysates were prepared with 200 μl Passive Lysis buffer (Promega, Madison, WI, USA). Aliquots of 20 μl of cleared lysate were assayed for luciferase activity with the Dual-luciferase^®^ reporter assay system (Promega).

### GST pull down assay

Three micrograms of GST or GST-CK2α was immobilized on 20 μl of Glutathione-Sepharose^TM^ 4B resin and incubated overnight at 4 °C with cell lysates from HEK293 cells transfected with HA-MZF1. After washing with PBS buffer, bound proteins were eluted by heating at 100 °C for 5 min with 2 × Laemmli sample buffer and resolved by 12% SDS-PAGE followed by western blot analysis.

### Immunoprecipitation

Cells were collected and lysed in 1 ml immunoprecipitation lysis buffer (50 mM Tris-HCl [pH 7.4], 150 mM NaCl, 0.5% NP-40) with complete protease inhibitor cocktail (Roche Diagnostics). The cell lysates were precleared and then incubated with the indicated antibodies for 1 h at 4 °C. The complexes were precipitated with Protein A/G-Sepharose beads (Santa Cruz Biotechnology Inc.), washed, and resuspended in 40 μl SDS loading buffer. Non-immune mouse IgG or non-immune rabbit IgG (Santa Cruz Biotechnology Inc.) served as a negative control.

### In vitro kinase assay

To document MZF1 phosphorylation by CK2, HA-tagged MZF1 expressed in HEK293 cells was immunoprecipitated and used as the substrate. Immunoprecipitated MZF1 and recombinant active human CK2α (ATGen Ltd, Seongnam-si, Gyeonggi-do, Korea) were incubated in a final volume of 50 μl kinase reaction buffer (10 μl of 5 × kinase buffer, 10 μl magnesium/ATP cocktail solution [90 μl of 75 mM MgCl_2_/500 mM ATP and 10 μl (100 μCi) [γ-^32^P]-ATP]) for 20 min at 30 °C. To measure intracellular CK2 activity, an in vitro kinase assay was performed as described previously with slight modification^29^. Bacterially expressed 3 μg of GST-CS (CK2 Substrate; GST-RRRDDDSDDD) was incubated with glutathione-Sepharose 4B beads for 60 min and washed twice with kinase buffer (4 mM MOPS [pH 7.2], 5 mM β-glycerophosphate, 1 mM EGTA, 200 μM sodium orthovanadate, and 200 μM DTT). The beads were then incubated with 100 μg of cell lysates in a final volume of 50 μl of kinase reaction buffer (10 μl of 5 × kinase buffer, 10 μl magnesium/ATP cocktail solution [90 μl of 75 mM MgCl_2_/500 mM ATP and 10 μl (100 μCi) [γ-^32^P]-ATP]) for 20 min at 30 °C. Reactions were stopped by washing twice with 1 × kinase buffer. Samples were resuspended with 30 μl of 2 × SDS sample loading buffer, subjected to 12% SDS-PAGE followed by staining with Coomassie brilliant blue, and dried on Whatman papers. Incorporation of ^32^P was detected by autoradiography.

### Mass spectrometry

To obtain MZF1 protein phosphorylated by CK2, in vitro kinase assays were performed with GST-MZF1 protein and recombinant active CK2α. Reactions were terminated by washing four times with kinase buffer. Samples were resuspended in 40 μl 5 × SDS sample loading buffer and boiled for 5 min. After electrophoresis, SDS polyacrylamide gels were stained with G250 Coomassie blue. GST-MZF1 was excised and digested in-gel with sequencing-grade, modified trypsin (Promega). In brief, the GST-MZF1 spot was excised from the gel, placed in a polypropylene tube, and washed four to five times with 150 μl of 1:1 acetonitrile/25 mM ammonium bicarbonate, pH 7.8. The gel slice was dried in a Speedvac concentrator and then rehydrated in 30 μl of 25 mM ammonium bicarbonate, pH 7.8, containing 20 ng of trypsin. After incubation at 37 °C for 20 h, the liquid was transferred to a new tube. Tryptic peptides remaining in the gel matrix were extracted for 40 min at 30 °C with 20 μl of 50% (v/v) aqueous acetonitrile containing 0.1% (v/v) formic acid. The combined supernatants were evaporated in a Speedvac concentrator and dissolved in 8 μl of 5% (v/v) aqueous acetonitrile solution containing 0.1% (v/v) formic acid for mass spectrometric analysis. The resulting tryptic peptides were separated and analyzed by reverse-phase capillary HPLC directly coupled to a Finnigan LCQ ion-trap mass spectrometer (LC-MS/MS). A 0.1 × 20-mm trapping column and a 0.075 × 130-mm resolving column were packed with Vydac 218 MS low trifluoroacetic acid C18 beads (5 μm in size, 300 Å in pore size; Vydac, Hesperia, CA) and placed in-line. The peptides were bound to the trapping column for 10 min with 5% (v/v) aqueous acetonitrile containing 0.1% (v/v) formic acid. The bound peptides were then eluted with a 50-min gradient of 5–80% (v/v) acetonitrile containing 0.1% (v/v) formic acid at a 0.2 μl/min flow rate. The full mass scan range mode was *m*/*z* = 450–2000 Da for tandem mass spectrometry. After the charge state of an ion was determined on the zoom scan, product ion spectra were acquired in MS/MS mode with relative collision energy of 55%. The individual spectra from MS/MS were processed with TurboSEQUEST software (Thermo Quest, San Jose, CA). The generated peak list files were used to query either the MSDB database or NCBI with the MASCOT program (http://www.matrixscience.com). Modifications of methionine and cysteine, peptide mass tolerance at 2 Da, MS/MS ion mass tolerance at 0.8 Da, allowance of missed cleavage at 2, and charge states (+1,+2, and +3) were all considered. Only significant hits by MASCOT probability analysis were initially considered.

### Site-directed mutagenesis of MZF1 and MZF1 binding site on *N-cadherin* promoter

Site-directed mutagenesis of *MZF1* or of the MZF1-binding site in the *N-cadherin* promoter was performed with a QuikChange site-directed mutagenesis kit (Stratagene). All mutant constructs were confirmed by DNA sequencing. The following mutagenic primer pairs were used to generate mutants: MZF1S27A: forward, 5′-TAGAGGACGCTGAGGAGGAG-3′; reverse, 5′-CTCCTCCTCAGCGTCCTCTA-3′. MZF1S27E: forward, 5′-TAGAGGACGAGGAGGAGGAG-3′; reverse, 5′-CTCCTCCTCCTCGTCCTCTA-3′. For mutations in the distal MZF1-binding site (TCCCCA to TCTTCA) in pNcad-667 (pNcad-667mt): forward, 5′-TTCCCCCGCCCCCTCTTCAGCTCCTTGATC-3′; reverse, 5′-GATCAAGGAGCTGAAGAGGGGGCGGGGGAA-3′.

### Chromatin immunoprecipitation (ChIP) assay

The in vivo molecular interaction between MZF1 and the *N-cadherin* promoter was analyzed by ChIP assay. HCE4 cells were transfected with pSG5-HA or pSG5-HA-MZF1 using Lipofectamine Plus (Invitrogen Co.). For the ChIP assay, chromatin was first isolated as follows. Approximately 2 × 10^9^ cells were treated with PBS containing 1% formaldehyde for 10 min, washed twice with PBS, and then incubated with 100 mM Tris (pH 9.4) and 10 mM DTT at 30 °C for 15 min. Cells were then washed twice with PBS and resuspended in 600 μl of Sol A buffer (10 mM HEPES [pH 7.9], 0.5% NP-40, 1.5 mM MgCl_2_, 10 mM KCl, 0.5 mM DTT) by pipetting. After a short spin (5 min at 3,000 rpm), the pellets were resuspended in Sol B buffer (20 mM HEPES [pH 7.9], 25% glycerol, 0.5% NP-40, 0.42 M NaCl, 1.5 mM MgCl_2_, and 0.2 mM EDTA) containing protease inhibitors followed by vigorous pipetting in order to extract the nuclear proteins. After centrifugation at 13,000 rpm for 30 min, the nuclear pellets were resuspended in immunoprecipitation buffer (1% Triton X-100, 2 mM EDTA, 20 mM Tris-HCl [pH 8.0], 150 mM NaCl, and protease inhibitors) and sonicated to break the chromatin into fragments with an average length of 0.5–1 kb. ChIP analysis of MZF1 binding to the *N-cadherin* promoter was performed with an antibody against HA or a nonspecific IgG Primers used were forward, 5′-GCTGCATCCCTCGCGGCTTC-3′, and reverse, 5′-GACAAATAGCGGGCCGCGGA-3′. To confirm MZF1 binding to a distal MZF1-binding site, HCE4 cells were transfected with pNcad-667 or pNcad-667mt with pSG5-HA or pSG5-HA-MZF1. Primers used were forward: sequences in pGL3-basic; reverse, 5′-CGAAGAGCCGGAGGAGGCGG-3′.

### Generation of stable MKD cells

shRNA-mediated knockdown of *MZF1* was performed using the HuSH-plasmid system (Origene Technologies Inc., Rockville, MD, USA). The shRNA sequences tested were as follows: Sequence #1: CCTGTCATGGTGAAGCTAGAGGACTCTGA. Sequence #2: TGCGATGTATGTGGCAAGGTGTTCAGCCA. Sequence #3: AGCATCTCCGCAGGTCCAGGTAGTGTAAG. Sequence #4: GGTTACAGAGGACTCAGATTTCCTGGAGT. We validated all constructs individually and found the #4 was the most effective construct for MKD (Supplementary Figure 1). After that, we used #4 construct for MKD. HCE4 cells were transfected with pRS-shMZF1#4 followed by treatment with puromycin (2 μg/ml) for selection, and TE2-CK2α cells were transfected with pGFP-B-RS-shMZF1#4 (Origene Technologies Inc.) followed by treatment with puromycin (1 μg/ml) and blasticidin (6 μg/ml) for selection.

### RNA isolation and reverse-transcription PCR (RT-PCR)

RNA was isolated from cultured cells with TRIzol (Invitrogen Co.) reagent. cDNA was synthesized with SuperScript II reverse transcriptase (Invitrogen Co.) and oligo-dT primers. PCR reactions were performed with 2 μl cDNA and a pair of primers specific for each gene. The sequences of the RT-PCR primers were: *N-cadherin*: forward, 5′-CACCCAACATGTTTACAATCAACAATGAGA-3′; reverse, 5′-CTGCAGCAACAGTAAGGACAAACATCCTAT-3′. *MZF1*: forward, 5′-CGCAGGTCCAGGTAGTGTAA-3′; reverse, 5′-ACTCTCCGATGCTCTTCCAG-3′.

### Quantitative real-time (qRT) PCR analysis

qRT-PCR was performed using Power SYBR Green PCR Master Mix (Applied Biosystems, Carlsbad, CA, USA). All qRT-PCR performed using SYBR Green was conducted at 50 °C for 2 min, 95 °C for 10 min, and then 40 cycles of 95 °C for 15 s and 60 °C for 1 min. The specificity of the reaction was verified by melt curve analysis. *Glyceraldehyde-3-phosphate dehydrogenase* (*GAPDH*) was used as a normalization control. The sequences of the qRT-PCR primers were: *MZF1* forward, 5′-GGGCCTGCAGGTGAAAGAG-3′; reverse, 5′-GGCAGCTAGAGGCCCAGACT-3′, *GAPDH* forward, 5′-GGCATCCTGGGCTACACTGA-3′; reverse, 5′-GAGTGGGTGTCGCTGTTGAA-3′.

### Cell proliferation assay

Cells were counted at 24 and 48 h after seeding, using an ADAM automatic Cell Counter (NanoEnTek, Inc., Seoul, Korea). Data represent the mean ± SD of one experiment performed in triplicate. Similar results were obtained from two independent experiments.

### Cell migration and invasion assay

The cell migration assay was performed with the OrisTM Cell Migration Assay kit (Platypus Technologies, LLC, Madison, WI, USA). Migration was quantified by measuring the cell surface area with ImageJ. Invasion was determined using 24-well chemotaxis chambers (Corning cell culture inserts, 8 μm pore size) with membranes pre-coated with Matrigel Basement Membrane Matrix (BD Biosciences, CA, USA). Complete medium with 10% FBS served as a chemoattractant in the bottom chamber and 4 × 10^5^ cells per ml cells were incubated for 48 h. Invaded cells at the bottom surface of the membrane were stained with 1% sulforhodamine B (SRB) for 5 min, dried and photographed. Bound dye was eluted with 10 mM Tris-HCl (pH 7.0). The ability of cell invasion was reflected by measuring the absorbance at 510 nm in a microplate reader.

### Statistical analysis

Statistical comparisons of groups were made using a Student’s *t* test, and *P* < 0.05 was considered significant. The Pearson correlation test was performed using R 3.0.

## Electronic supplementary material

Fig. S1. Determining shRNA functions through qRT-PCR

Fig.S2. Cell migration assay with the OrisTM Cell Migration Assay kit

Fig.S3. Effect of CK2 on the expression level of Snail, β-catenin, and Axin2
