# Supplementary figures and images for: Phosphorylation-dependent stabilization of MZF1 upregulates N-cadherin expression during protein kinase CK2-mediated epithelial-mesenchymal transition
Source: Oncogenesis. 2018 Mar 13;7(3):27. doi: 10.1038/s41389-018-0035-9 (PMC5852951; doi:10.1038/s41389-018-0035-9)

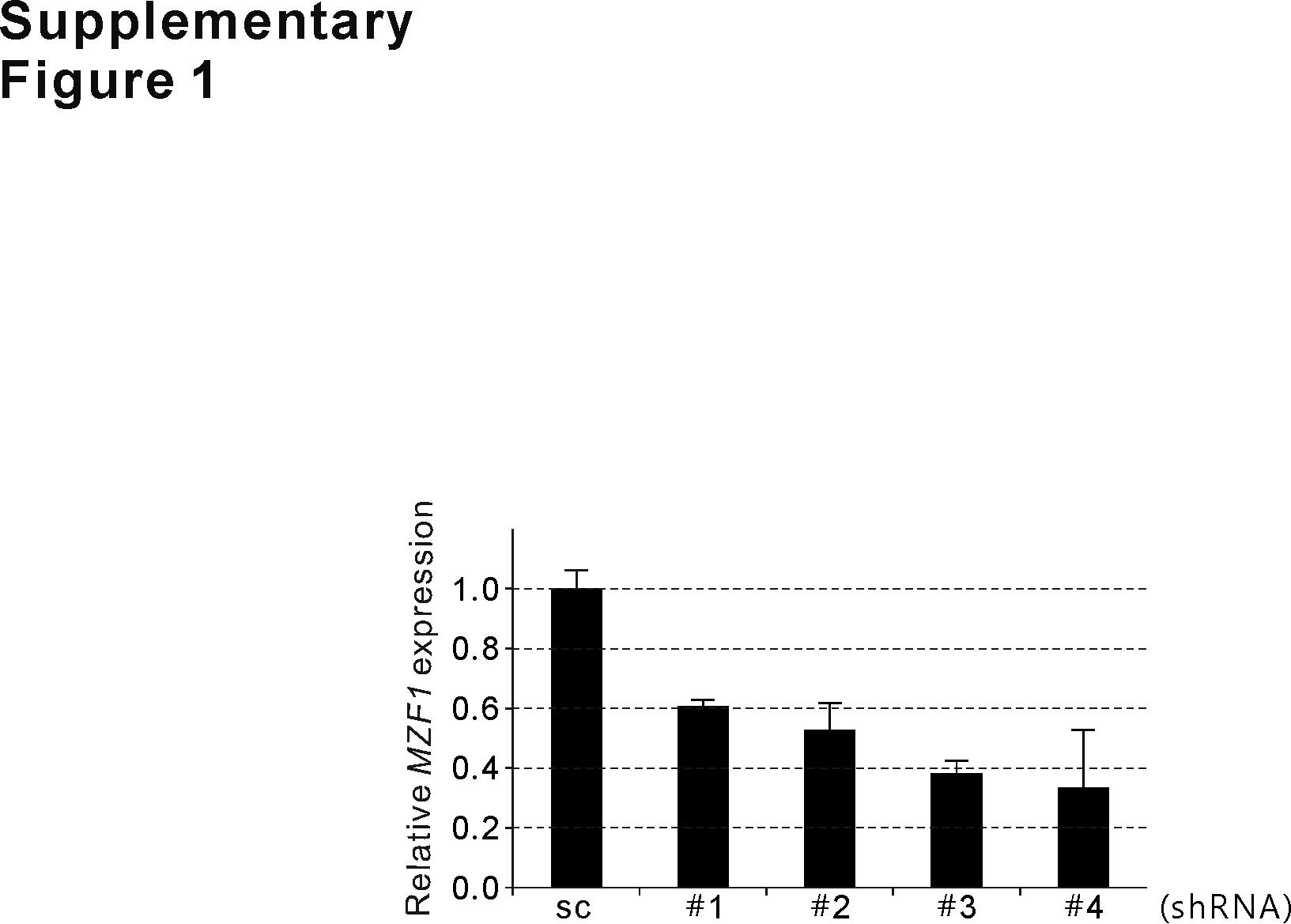

Supplement: Supplementary file 1 — Fig. S1. Determining shRNA functions through qRT-PCR [file 41389_2018_35_MOESM1_ESM.tif]

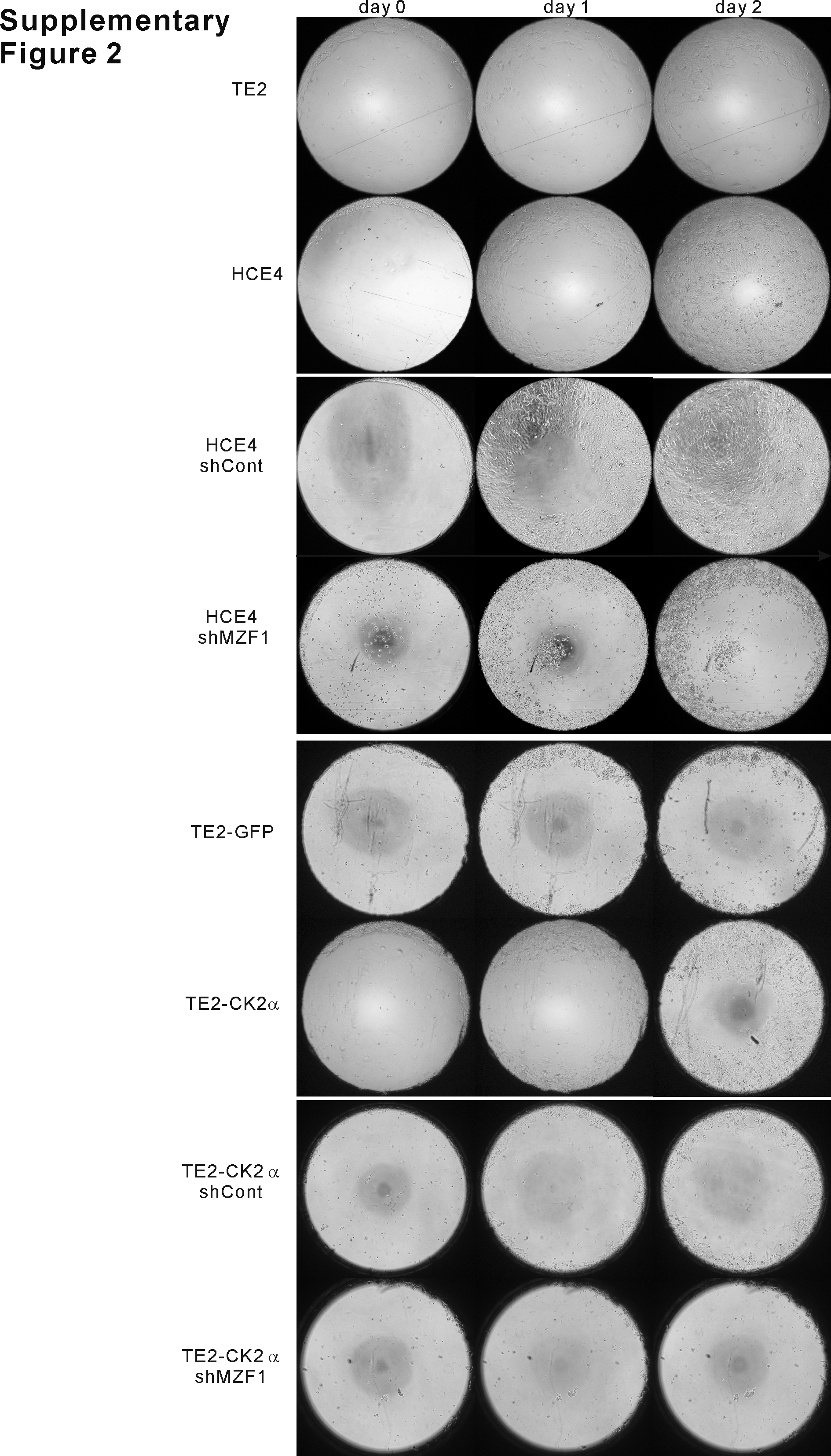

Supplement: Supplementary file 2 — Fig.S2. Cell migration assay with the OrisTM Cell Migration Assay kit [file 41389_2018_35_MOESM2_ESM.tif]

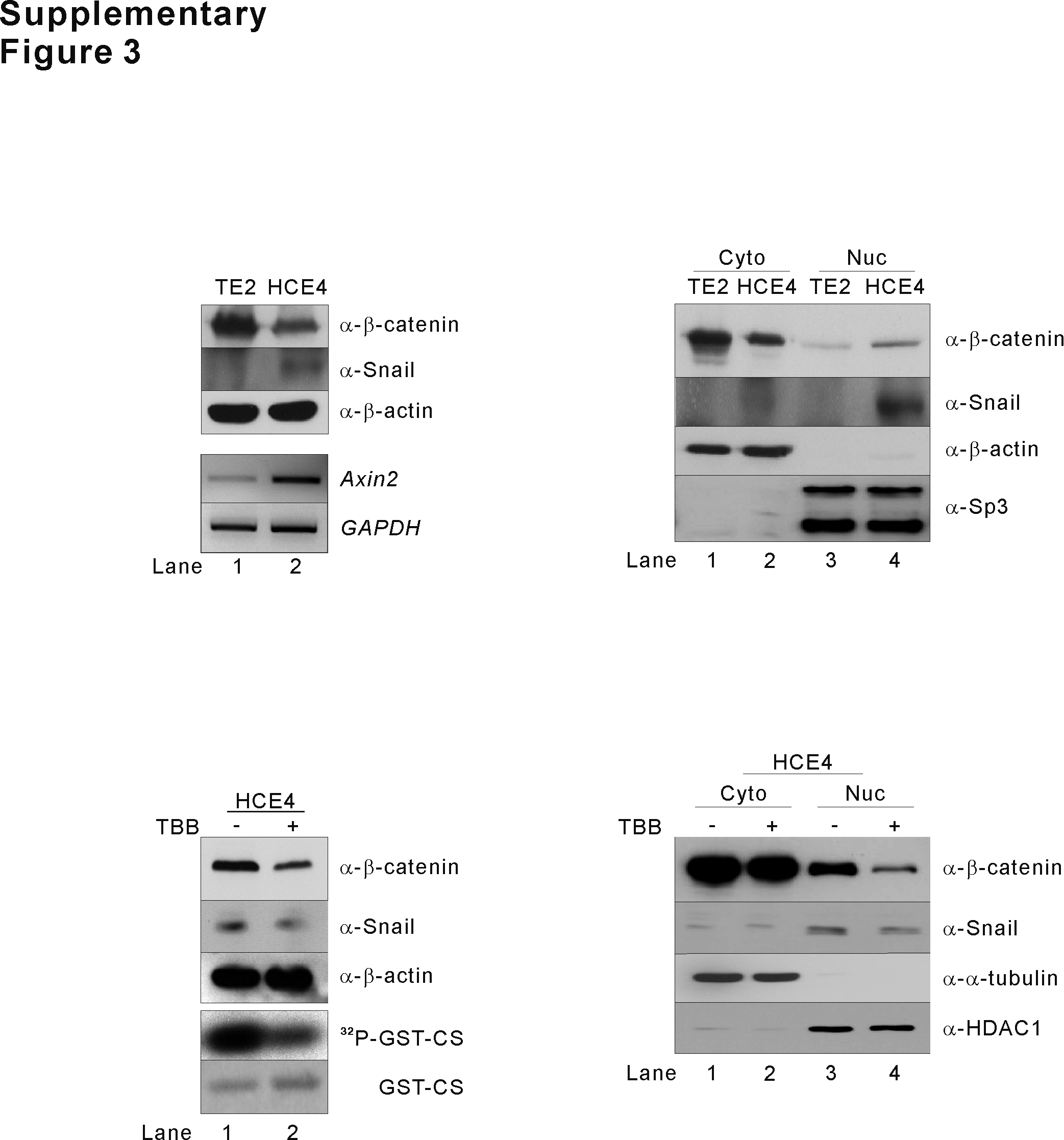

Supplement: Supplementary file 3 — Fig.S3. Effect of CK2 on the expression level of Snail, β-catenin, and Axin2 [file 41389_2018_35_MOESM3_ESM.tif]
